# Factors associated with increased pancreatic enzymes in septic patients: a prospective study

**DOI:** 10.1186/s40560-017-0243-y

**Published:** 2017-07-14

**Authors:** Anis Chaari, Karim Abdel Hakim, Nevine Rashed, Kamel Bousselmi, Vipin Kauts, Mahmoud Etman, William Francis Casey

**Affiliations:** 1 Critical Care Department, King Hamad University Hospital, Al Muharaq, Bahrain; 2 Gastroenterology Department, King Hamad University Hospital, Al Muharaq, Bahrain

**Keywords:** Amylase, Lipase, Septic shock, Prognosis

## Abstract

**Background:**

The perfusion of splanchnic organs is deeply altered in patients with septic shock. The aim of the study is to identify the predictive factors of septic shock-induced increase of serum lipase and amylase and to assess and evaluate its prognostic impact.

**Methods:**

We conducted a prospective observational study. All adult patients admitted with septic shock were eligible for our study. Serum lipase and amylase were measured on admission. Patients with and those without increased pancreatic enzymes were compared. Predictive factors of pancreatic insult identified by the univariate analysis were integrated in a stepwise multivariate analysis. Odds ratios (OR) with the 95% confidence interval (CI) were calculated accordingly. Second, the sensitivity and the specificity of amylase and lipase to predict intensive care unit (ICU) mortality were identified through the Receiver Operator Curve.

**Results:**

Fifty patients were included. Median [quartiles] age was 68.5 [58–81] years. The APACHE II score was 26 [20–31]. Twenty-three patients (46%) had increased serum amylase and/or serum lipase. Diabetes mellitus (OR = 16; 95% CI [1.7–153.5]; *p* = 0.016), increased blood urea nitrogen (OR = 1.12; 95% CI [1.02–1.20], *p* = 0.016), and decreased C-reactive protein (OR = 0.97; 95% CI [0.96–0.99]; *p* = 0.027) were identified as independent factors predicting increased pancreatic enzymes. Twenty patients (40%) died in the ICU. Neither serum amylase level nor serum lipase level was significantly different between survivors and non-survivors (respectively 49 [27.7–106] versus 85.1 [20.1–165] UI/L; *p* = 0.7 and 165 [88–316] versus 120 [65.5–592] UI/L; *p* = 0.952).

**Conclusion:**

Increase of pancreatic enzymes is common in patients with septic shock. Diabetes and impaired renal function are predictive of increased pancreatic enzymes. Such finding does not carry any negative prognostic value.

## Background

Septic shock is a common cause of admission to the intensive care units (ICUs) [1, 2]. Despite subsequent improvement of management modalities and worldwide awareness of updated guidelines, it still carries a high mortality ranging from 30 to 60% [1, 2]. This high mortality is mainly related to the early onset of multiorgan failure due to increased demand and decreased supply of oxygen [3, 4]. Several studies have highlighted that renal, hepatic, and hematological dysfunctions in patients with septic shock are associated with poor outcome [4–6]. The aim of the study is to identify the predictive factors of septic shock-induced increase of serum lipase and amylase and to assess and evaluate its prognostic impact.

## Methods

### Study design

We conducted a prospective observational study in the intensive care unit of King Hamad University Hospital (KHUH) between 1 October 2015 and 31 May 2016. The study was approved by the ethics committee of KHUH.

### Patients

All the adult patients admitted with septic shock were eligible for inclusion in the study. We excluded patients with previous pancreatic disease, those with history of alcohol abuse, and those with biliary tract infections. Septic shock was defined according to the third international definition for sepsis and septic shock [7]. Therefore, serum lactate level was checked on admission and Sequential Organ Failure Assessment (SOFA) score was calculated [8]. The Increase of pancreatic enzymes was defined by a serum amylase level higher than 85 UI/L and/or serum lipase level higher than 286 UI/L. The diagnosis of acute pancreatitis was considered if two criteria among the following were met: (1) abdominal pain, (2) increased serum pancreatic enzymes (higher than threefold the upper limit of the normal range), (3) imaging findings suggesting acute pancreatitis [9].

### Data collection and protocol of the study

For all the included patients, we recorded demographic characteristics (age, gender, comorbidities), cause of admission to the ICU, clinical findings on admission to the ICU (systolic and diastolic blood pressure, heart rate), laboratory results on admission to the ICU (arterial lactate, complete blood cell count, C-reactive protein, procalcitonin, renal function tests), and therapeutic interventions during the ICU stay (mechanical ventilation, vasopressors, antibiotics, nutritional support, and continuous renal replacement therapy). The source of sepsis and the identified microorganisms were also recorded. The severity on admission was assessed by calculating the Acute Physiology and Chronic Health Evaluation II (APACHE (II)) score [10].

Pancreatic enzymes (serum amylase and lipase) were checked on admission. Patients with increased pancreatic enzymes underwent either abdominal ultrasound or contrast-enhanced abdominal computed tomography.

We also recorded the duration of mechanical ventilation, the length of ICU stay, and the outcome (death/survival).

### Statistics

Data were expressed as percentages for the qualitative variables and means ± standard deviation (SD) or median [quartiles] as appropriate for the quantitative variables. Two groups were compared: those with increased pancreatic enzymes (increased pancreatic enzymes (+)) and those with normal pancreatic enzymes levels (increased pancreatic enzymes (−)). The normal distribution of the quantitative variables was checked by Shapiro-Wilk test. The qualitative variables were compared by using the chi-square test or Fisher’s exact test as appropriate. The quantitative variables were compared by using *t* test or Mann-Whitney test as appropriate. All the factors identified by the univariate analysis as statistically associated to the increase of pancreatic enzymes were integrated in a stepwise multivariate analysis. Odds ratios (OR) were therefore calculated with the corresponding 95% confidence interval (95% CI)

Second, the sensitivity and the specificity of pancreatic enzymes in predicting ICU mortality were assessed by constructing the Receiver Operator Curves (ROC). The area under curve was calculated for each parameter with the respective 95% confidence interval.

## Results

### Baseline characteristics

Fifty patients were included in the study. Median [quartiles] age was 68.5 [58–81] years. Sex ratio (M/F) was 1.2. The APACHE (II) score calculated within the first 24 h was 26 [20–31]. Median SOFA score on admission was 7 [6–10]. Thirty patients (60%) were diabetic, and 32 patients (64%) have previous history of hypertension. On admission to the intensive care unit (ICU), median systolic blood pressure was 88.5 [77–106] mmHg and median diastolic blood pressure was 50 [40–60] mmHg. Median heart rate was 102 [76–120] beats per minute. The source of sepsis was pneumonia in 20 patients (40%), urinary tract infection in 11 patients (22%), soft tissue infection in 9 patients (18%), and abdominal infection in 10 patients (20%). Cultures were positive in 24 patients (48%). The identified microorganisms were gram-positive cocci in 5 patients (10%) and gram-negative bacilli in 19 patients (38%).

### Pancreatic function

Twenty-three patients (46%) had increased serum amylase and/or serum lipase. Median serum amylase level was 61 [24—139] UI/L. Nineteen patients (38%) had increased serum amylase, and 7 patients (14%) had a serum level higher than 3 times the upper limit. Median serum lipase level was 147 [77–316] UI/L. Thirteen patients (26%) had increased serum lipase level, and 5 patients (10%) had a level higher than 3 times the upper limit. All patients with increased pancreatic enzymes underwent imaging tests. Abdominal computed tomography was performed for 6 patients (12%) whereas abdominal ultrasound was performed for 17 patients (34%). Only one patient (2%) was diagnosed as acute pancreatitis Balthazar B whereas all the other patients had unremarkable imaging investigations.

### Management

All our patients required vasopressor support on admission to the ICU. Median norepinephrine dose was 0.4 [0.1–1.5] microgram/kg/min. Median duration of norepinephrine infusion was 3 [2–5] days. Mechanical ventilation was started on admission to the ICU in 32 patients (64%). Median duration of mechanical ventilation was 5 [2–14] days. All of our patients were started on enteral feeding within the first 24 h of admission and none of them received parenteral nutrition. Continuous renal replacement therapy was required for 24 patients (48%) and was started within 2 [1–3] days after admission.

### Predictive factors of sepsis-induced increase of pancreatic enzymes

Univariate analysis showed that the incidence of diabetes mellitus as a morbidity was significantly higher in increased pancreatic enzymes (+) group (78.3 versus 44.4%; *p* = 0.015). The APACHE (II) score was also significantly higher in this group of patients (Table 1). The analysis of the biological findings on admission showed that patients with increased pancreatic enzymes had significant increase in renal function tests and lower serum C-reactive protein level (Table 2).

**Table 1:** Comparison of the baseline characteristics between patients with and those without increased pancreatic enzymes

Parameters	Increased pancreatic enzymes (+) (*N* = 23)	Increased pancreatic enzymes (−) (*N* = 27)	*p*
Age (years [quartiles])	71 [60–82]	67 [57–79]	0.190
Gender (M/F)	13/10	14/13	0.714
Hypertension (*N*/%)	17/73.9	15/55.6	0.178
Diabetes mellitus (*N*/%)	18/78.3	12/44.4	0.015
APACHE (II) ([quartiles])	28 [25–33]	24 [19–29]	0.032
SOFA score ([quartiles])	9 [6–12.3]	6 [5–9]	0.100

*APACHE (II)* Acute Physiology and Chronic Health Evaluation II, *SOFA* Sequential Organ Failure Assessment

**Table 2:** Comparison of the laboratory findings on admission between patients with and those without increased pancreatic enzymes

Parameters	Increased pancreatic enzymes (+) (*N* = 23)	Increased pancreatic enzymes (−) (*N* = 27)	*p*
Amylase (UI [quartiles])	153 [97.2–445]	26 [16.6–44.7]	0.029
Lipase (UI [quartiles])	337 [146–744]	101 [68.5–166.3]	0.027
Lactate (mmol/L [quartiles])	3.9 [2.5–5.3]	3.4 [2–4.1]	0.228
CRP (mg/L [quartiles])	114.5 [55.5–196.8]	217 [120.5–281]	0.017
Procalcitonin (ng/mL [quartiles])	38.8 [2.2–97.6]	5.1 [1.8–46.6]	0.575
BUN (mmol/L [quartiles])	16.9 [9–33]	11.1 [5.4–17.4]	0.038
Creatinine (μmol/L [quartiles])	204 [131.3–331.8]	146 [84–209]	0.038

*CRP* C-reactive protein, *BUN* blood urea nitrogen

In multivariate analysis, independent factors predicting sepsis-induced increase of pancreatic enzymes were diabetes mellitus (OR = 16; 95% CI [1.7–153.5]; *p* = 0.016), increased blood urea nitrogen (OR = 1.12; 95% CI [1.02–1.20]; *p* = 0.016), and decreased C-reactive protein (OR = 0.97; 95% CI [0.96–0.99]; *p* = 0.027) (Table 3).

**Table 3:** Multivariate analysis of factors predicting sepsis-related increase of pancreatic enzymes

Factors	OR	*p*	95% CI	
			Min	Max
APACHE (II)	1.10	0.182	0.90	1.20
Diabetes mellitus	16	0.016	1.70	153.50
C-reactive protein	0.97	0.027	0.96	0.99
Blood urea nitrogen	1.12	0.016	1.02	1.20
Creatinine	1	0.929	0.99	1.10

*APACHE (II)* Acute Physiology and Chronic Health Evaluation II

### Outcome and prognostic factors

**Fig. 1:**
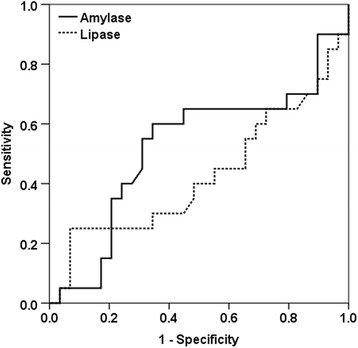
ROC for sensitivity and specificity of serum amylase and serum lipase to predict ICU mortality. AUC for serum amylase 0.52 95% CI [0.35–0.7]. AUC for serum lipase 0.57 95% CI [0.40–0.74]. *ROC* Receiver Operator Curve, *AUC* area under curve, *CI* confidence interval

Twenty patients (40%) died in the ICU. Median ICU length of stay was 6 [3–12] days. There was no statistically significant difference between survivors and non-survivors regarding the baseline characteristics except for the APACHE (II) score which was significantly higher in non-survivors group (Table 4). Moreover, the rate of patients requiring mechanical ventilation support and/or renal replacement therapy was significantly higher in the non-survivors group (Table 5). Laboratory findings were comparable between the two studied groups. Neither serum amylase level nor serum lipase level was significantly different between survivors and non-survivors (respectively 49 [27.7–106] versus 85.1 [20.1–165] UI/L; *p* = 0.7 and 165 [88–316] versus 120 [65.5–592] UI/L; *p* = 0.952). Both pancreatic enzymes had poor value to predict ICU mortality (Fig. 1).

**Table 4:** Comparison of baseline characteristics between survivors and non-survivors

Parameters	Survivors (*N* = 30)	Non-survivors (*N* = 20)	*p*
Age (years [quartiles])	66.5 [56.8–79.8]	70.5 [61.8–81]	0.220
Gender (M/F)	16/14	11/9	0.910
Diabetes mellitus (*N*/%)	17/56.7	13/65	0.556
Hypertension (*N*/%)	18/60	14/70	0.470
APACHE (II) ([quartiles])	21 [16.8–29]	29.5 [27–33.5]	<0.001
Leucocytes (mm^3^ [quartiles])	11,820 [8740–17,375]	19,380 [8805–26,125]	0.060
Hemoglobin (g/dL [quartiles])	10.6 [8.6–12.5]	10.4 [8.4–12.2]	0.400
Platelets count (g/L [quartiles])	233.5 [163.3–376.8]	279 [158.3–435]	0.318
CRP (mg/L [quartiles])	146 [79.9–271]	174 [129–226.5]	0.795
Lactate (mmol/L [quartiles])	3.7 [2.5–5.1]	3.2 [2.2–4.1]	0.707

*APACHE (II)* Acute Physiology and Chronic Health Evaluation II, *CRP* C-reactive protein

**Table 5:** Comparison of the therapeutic management between survivors and non-survivors

Parameters	Survivors (*N* = 30)	Non-survivors (*N* = 20)	*p*
MV (*N*/%)	12/40	20/100	<0.001
Duration of MV (days [quartiles])	6 [2.3–18.8]	3.5 [2–12.5]	0.344
CRRT (*N*/%)	10/33.3	14/70	0.011
NE (*N*/%)	30/100	20/100	1
NE dose (mcg/kg/min [quartiles])	0.2 [0.09–0.42]	2 [0.7–2]	<0.001

*MV* mechanical ventilation, *CRRT* continuous renal replacement therapy, *NE* norepinephrine

## Discussion

Our study shows that the increase of pancreatic enzymes is common in patients with septic shock. In fact, 46% of the included patients had increased serum amylase and/or lipase. Previous studies have reported that the incidence of exocrine pancreatic dysfunction in critically ill patients ranges between 14 and 80% [6, 11, 12]. This wide range is due to the underlying diseases as the highest levels were recorded in surgical and trauma patients [13, 14]. However, epidemiological data regarding pancreatic dysfunction in patients with septic shock are lacking.

The increase of pancreatic enzymes in critically ill patients is mainly due to ischemic insults induced by prolonged hypotension [11, 15]. In fact, experimental studies have shown that the pancreas is extremely sensitive to hypoxia and that peripheral lobule necrosis can occur within 40 min of hypotension [16].

Other factors such as the activation of the coagulation cascade, cellular apoptosis, oxidative stress, and disturbed lipid profile have been reported as possible causes of sepsis-related increase of pancreatic enzymes [6]. Therefore, the involvement of these factors in the physiopathology of pancreatic insult may explain the occurrence of this complication in patients without significant hemodynamic compromise [6, 17]. Our study also showed that the impairment of renal function was significantly associated with sepsis-related increase of pancreatic enzymes. Previous studies have reported that acute kidney injury is a common complication of acute pancreatitis [18, 19]. Hypertriglyceridemia has been reported as a major factor responsible of acute kidney injury [18, 19].

Second, our study showed that independent factors predicting sepsis-related increase of pancreatic enzymes are diabetes mellitus and acute kidney injury. Previous studies have reported that patients with type 2 diabetes mellitus have two- to threefold higher risk of acute pancreatitis [20–23]. This higher risk has been confirmed even after adjusting for confounding factors such as alcohol abuse and obesity [21, 22]. The underlying mechanisms are still not fully elucidated but may involve disturbed lipid metabolism or drug toxicity [23]. Our study also showed that increased renal function tests were significantly associated with increased pancreatic enzymes. Previous studies showed that acute kidney injury is common in patients with acute pancreatitis [18, 19]. Hypertriglyceridemia has been reported as a major cause of renal dysfunction in this group of patients [18, 19].

The multivariate analysis also revealed that decreased C-reactive protein independently predicts increased pancreatic enzymes. Available data assessing the usefulness of this biomarker in predicting pancreatic necrosis are scarce. However, a systematic review showed that C-reactive protein, as well as procalcitonin and lactate deshydrogenase, is not reliable to predict pancreatic injury [24]. Further studies are required to confirm the correlation between decreased CRP and increased pancreatic enzymes and to investigate the underlying mechanisms that might explain this relationship.

Whether the increase of pancreatic enzymes carries per se a negative prognostic value or not is still a matter of debate. Manjuck et al. [12] reported that the level of pancreatic enzymes was not correlated to the mortality. However, the ICU length of stay and the duration of mechanical ventilation were significantly higher in patients with increased pancreatic enzymes. Similarly, Subramanian et al. [14] reported that the level of serum lipase was positively correlated with the incidence of organ failure in critically ill trauma patients. In patients with septic shock, Pizzelli et al. [15] reported that all the 21 included patients had increased pancreatic enzymes but none of them had clinical or radiological abnormalities suggesting acute pancreatitis. Our results corroborate these findings as only one of our patients had confirmed acute pancreatitis.

To the best of our knowledge, our study is the first to identify the predictive factor of sepsis-induced increase of pancreatic enzymes and to assess its prognostic value. However, several limitations should be mentioned. First, our study has a small sample size. Second, the involvement of the renal impairment in the rise of pancreatic enzymes needs to be investigated. In fact, the increase of amylase and lipase in patients with acute kidney injury might be related to a delayed clearance of these enzymes. In this regard, Seno et al. [25] compared in an observational study the serum level of 6 pancreatic markers between 2 groups: 47 patients with impaired renal function and 24 healthy individuals. The authors reported that the serum levels of total amylase, pancreatic isoamylase (p-amylase), lipase, phospholipase A2, and elastase I were at least within 2.5-fold the upper limits of the normal ranges in patients with renal impairment The level was even higher (within 4.8-fold the upper limit) for trypsin(ogen). Finally, the diagnosis of acute pancreatitis was based on the levels of serum amylase and lipase. It has been shown that lipase is more specific than amylase for accurate diagnosis of acute pancreatitis [9]. Moreover, the alpha amylase fraction is more specific of pancreatic injury than total amylase. Yet only total amylase was measured in our study [9].

## Conclusion

The increase of pancreatic enzymes is common in patients with septic shock. Diabetes mellitus, increased urea, and low C-reactive protein independently predict raise in pancreatic enzyme. Our results suggest that such finding is not associated with worse outcome and therefore should not trigger any specific therapeutic intervention. Further studies are required to confirm our conclusions.

## Abbreviations

APACHE (II): Acute Physiology and Chronic Health Evaluation II
BUN: Blood urea nitrogen
CI: Confidence interval
CRP: C-reactive protein
CRRT: Continuous renal replacement therapy
ICU: Intensive care unit
MV: Mechanical ventilation
NE: Norepinephrine
OR: Odds ratio
ROC: Receiver Operator Curve
SD: Standard deviation

## References

[1] Angus DC, Linde-Zwirble WT, Lidicker J, Clermont G, Carcillo J, Pinsky MR (2001). Epidemiology of severe sepsis in the United States: analysis of incidence, outcome, and associated costs of care. Crit Care Med.

[2] Annane D, Aegerter P, Jars-Guincestre MC, Guidet B (2003). Current epidemiology of septic shock: the CUB-Rea Network. Am J Respir Crit Care Med.

[3] Angus DC, van der Poll T (2013). Severe sepsis and septic shock. N Engl J Med.

[4] Dellinger RP, Levy MM, Rhodes A, Annane D, Gerlach H, Opal SM (2013). Surviving sepsis campaign: international guidelines for management of severe sepsis and septic shock: 2012. Crit Care Med.

[5] Honore PM, Jacobs R, Hendrickx I, Bagshaw SM, Joannes-Boyau O, Boer W (2015). Prevention and treatment of sepsis-induced acute kidney injury: an update. Ann Intensive Care.

[6] Chaari A, Abdel Hakim K, Bousselmi K, Etman M, El Bahr M, El Saka A (2016). Pancreatic injury in patients with septic shock: a literature review. World J Gastrointest Oncol.

[7] Singer M, Deutschman CS, Seymour CW, Shankar-Hari M, Annane D, Bauer M (2016). The Third International Consensus Definitions for Sepsis and Septic Shock (Sepsis-3). JAMA.

[8] Vincent JL, Moreno R, Takala J, Willatts S, De Mendonça A, Bruining H (1996). The SOFA (Sepsis-related Organ Failure Assessment) score to describe organ dysfunction/failure. On behalf of the Working Group on Sepsis-Related Problems of the European Society of Intensive Care Medicine. Intensive Care Med.

[9] Working Group IAPAPAAPG (2013). IAP/APA evidence-based guidelines for the management of acute pancreatitis. Pancreatology.

[10] Knaus WA, Draper EA, Wagner DP, Zimmerman JE (1985). APACHE II: a severity of disease classification system. Crit Care Med.

[11] Denz C, Siegel L, Lehmann KJ, Dagorn JC, Fiedler F (2007). Is hyperlipasemia in critically ill patients of clinical importance? An observational CT study. Intensive Care Med.

[12] Manjuck J, Zein J, Carpati C, Astiz M (2005). Clinical significance of increased lipase levels on admission to the ICU. Chest.

[13] Takahashi M, Maemura K, Sawada Y, Yoshioka T, Sugimoto T (1980). Hyperamylasemia in critically injured patients. J Trauma.

[14] Subramanian A, Albert V, Mishra B, Sanoria S, Pandey RM (2016). Association between the pancreatic enzyme level and organ failure in trauma patients. Trauma Mon.

[15] Pezzilli R, Barassi A, Imbrogno A, Fabbri D, Pigna A, Morselli-Labate AM, Corinaldesi R, d'Eril GM (2011). Is the pancreas affected in patients with septic shock?—a prospective study. Hepatobiliary Pancreat Dis Int.

[16] Spormann H, Sokolowski A, Letko G (1989). Effect of temporary ischemia upon development and histological patterns of acute pancreatitis in the rat. Pathol Res Pract.

[17] Tribl B, Bateman RM, Milkovich S, Sibbald WJ, Ellis CG (2004). Effect of nitric oxide on capillary hemodynamics and cell injury in the pancreas during Pseudomonas pneumonia-induced sepsis. Am J Physiol Heart Circ Physiol.

[18] Wu C, Ke L, Tong Z, Li B, Zou L, Li W, Li N, Li J (2014). Hypertriglyceridemia is a risk factor for acute kidney injury in the early phase of acute pancreatitis. Pancreas.

[19] Nawaz H, Koutroumpakis E, Easler J, Slivka A, Whitcomb DC, Singh VP (2015). Elevated serum triglycerides are independently associated with persistent organ failure in acute pancreatitis. Am J Gastroenterol.

[20] Steinberg WM, Buse JB, Ghorbani MLM, Orsted DD, Nauck MA. Amylase, lipase, and acute pancreatitis in people with type 2 diabetes treated with liraglutide: results from the LEADER randomized trial. Diabetes care. 2017. 10.2337/dc16-2747 28476871

[21] Girman CJ, Kou TD, Cai B, Alexander CM, O'Neill EA, Williams-Herman DE, Katz L (2010). Patients with type 2 diabetes mellitus have higher risk for acute pancreatitis compared with those without diabetes. Diabetes Obes Metab.

[22] Noel RA, Braun DK, Patterson RE, Bloomgren GL (2009). Increased risk of acute pancreatitis and biliary disease observed in patients with type 2 diabetes: a retrospective cohort study. Diabetes care.

[23] Urushihara H, Taketsuna M, Liu Y, Oda E, Nakamura M, Nishiuma S (2012). Increased risk of acute pancreatitis in patients with type 2 diabetes: an observational study using a Japanese hospital database. PloS one.

[24] Komolafe O, Pereira SP, Davidson BR, Gurusamy KS (2017). Serum C-reactive protein, procalcitonin, and lactate dehydrogenase for the diagnosis of pancreatic necrosis. Cochrane Database Syst Rev.

[25] Seno T, Harada H, Ochi K, Tanaka J, Matsumoto S, Choudhury R (1995). Serum levels of six pancreatic enzymes as related to the degree of renal dysfunction. Am J Gastroenterol.

